# A Comparative Analysis of the Incidence of Pediatric Orbital Fractures Before and During the COVID-19 Pandemic in the Japanese Population

**DOI:** 10.7759/cureus.54166

**Published:** 2024-02-14

**Authors:** Steffani Krista Someda, Yasuhiro Takahashi

**Affiliations:** 1 Oculoplastic, Orbital and Lacrimal Surgery, Aichi Medical University Hospital, Nagakute, JPN

**Keywords:** sports injury, novel coronavirus, pediatric orbital fracture, pandemic restriction, coronavirus disease 2019

## Abstract

Introduction: The aim of this study is to compare data on the incidence of pediatric orbital fractures before the onset of the coronavirus disease 2019 (COVID-19) pandemic and during the period of the pandemic in the Japanese population.

Methods: This retrospective, single-center, observational study including 225 patients (226 sides) aged ≤ 18 years old diagnosed with orbital fracture was conducted in our institution from March 2017 to April 2023. The study compared the incidence of pediatric orbital fractures in the pre-pandemic period from March 2017 to March 2020 and during the pandemic from April 2020 to April 2023.

Results: The most common cause of injury was sports in both groups (137 sides, 60.6%), and the ratio of causes of injury (P = 0.610) or between outdoor and indoor sports (P = 1.000) was not statistically different between the groups. Although the daily rate of patient consults was lowest during the country’s state of emergency with priority preventative measures, the difference between pre-pandemic and pandemic was not statistically significant (P = 0.911).

Conclusion: Despite the restrictions mandated by the Japanese government during the COVID-19 pandemic, the physical activities of children did not significantly decline. Hence, the risk of pediatric orbital fractures remained the same.

## Introduction

The year 2020 has caused a worldwide shift in the economy and lifestyle due to the outbreak of coronavirus disease 2019 (COVID-19). The disease has affected many lives in every nation that the virus has spread into. This resulted in the urgent implementation of a nationwide restriction of human activities in order to control the transmission of the novel coronavirus from person to person. Seeing the fatal consequences of COVID-19 that plagued every news station all over the world, fear and paranoia afflicted families as they attempted to protect their children from the dreaded infection.

The Japanese government declared the first state of national emergency on April 7, 2020, in seven urban prefectures, eventually expanding to the entire country on April 16, 2020 [1,2]. In order to reduce the spread of COVID-19, schools were closed and people all over Japan were advised to stay at home. With the strict social distancing protocol implemented at this time, children were forbidden from engaging in sports and other recreational activities. Because of the nationwide restriction, it can be expected that sports-related injuries may have subsequently declined. And since the children are not going to school and have minimal interaction with the other children, it would also be expected that cases of physical assault would decrease. Imposing restrictions and social distancing measures have also prevented the adult population from going out. Therefore, the incidence of traffic accidents may have also decreased at this point in time.

A comparative study done in Italy during the height of the pandemic reported a decrease in ocular injuries in children and adolescents due to sports and fall incidents, from 354 patients in 2019 to only 112 in 2020, which may likely be caused by the drop in patients seeking emergency care at this period in time [3]. A similar trend was also found in another study involving adult patients [4]. In the United States, Wu et al. in their 2020 study likewise found a decrease in the number of emergency ophthalmic consultations but revealed a higher incidence of ocular injuries sustained while at home, which was mainly due to home improvement projects coupled with the lack of proper eye protection [5]. Although a recent study done in the USA reported a short-term increase in the incidence of ocular trauma during the summer months, causing a rebound in cases, there was still an overall decrease in sports-related injuries in 2020 due to the stay-at-home orders by the American government [6]. Interestingly, a study in India reported an increase in sports-related injuries from March to July 2020 due to an increase in recreational sports activity amidst the COVID-19 pandemic. However, there was still a significant decline in the total number of ocular trauma cases [7]. A previous cohort study done in Japan has also found a similar trend with a decrease in emergency consultations during the pandemic as compared to the period before the pandemic [1]. These previous studies have provided data on certain populations, but there is still very few data in the current literature with regard to the Japanese population and the epidemiological changes of pediatric orbital fractures associated with the COVID-19 pandemic.

Given the fact that children do not normally engage in home improvement projects or violence amongst family members, the impact of the COVID-19 pandemic on children has been particularly inconspicuous as compared to the adult, or even general, population. There is also the factor of having parents who desire nothing more than to protect their children from the detrimental effects of COVID-19. Furthermore, discipline is instilled in the Japanese culture at the earliest possible age, so adults and children alike can be expected to follow rules quite diligently. Nevertheless, it is also unlikely that parents would continue to withhold their children from playing or engaging in recreational activities to enjoy their childhood. As such, the question remains whether or not COVID-19 has made an impact on the incidence of ocular injuries in the pediatric population.

This study aims to compare data on the incidence of pediatric orbital fractures caused by sports injuries, falls, traffic accidents, and other etiologies prior to the onset of the COVID-19 pandemic and during the period of the pandemic. Moreover, the study aims to determine the most common cause of orbital fractures in children before and during the COVID-19 nationwide restriction imposed in Japan. The study can provide data that can be used to gain a better understanding of the epidemiological changes in pediatric orbital fracture cases associated with the COVID-19 pandemic.

## Materials and methods

This is a retrospective, observational study including all patients with orbital fractures who were referred to our service from March 2017 to April 2023. Patients living in Aichi, Mie, and Gifu prefectures have sought consultation with our service. Patients with concomitant orbital rim fracture (impure orbital fracture) and those with old orbital fracture were excluded from this study.

The data on age, sex, affected side, causes of injury, presence or absence of infraorbital nerve hypoesthesia, fields of binocular single vision (BSV) examined on the first visit, and surgery were collected. The study defined the “student” patients in Japan as patients aged ≤ 18 years (elementary school students, 7-12 years; junior high school students, 13-15 years; and high school students, 16-18 years). Patients aged six years or younger were also included because most children aged six years or younger go to nursery school or kindergarten in Japan. Causes of injury were classified as follows according to a previous study: sports, assault, falls, traffic accidents, and others [8]. Sports were subdivided into outdoor (baseball, soccer, rugby, etc.) and indoor sports (basketball, volleyball, boxing, etc.). The results of the field of BSV were classified into five categories (B1 to B5), according to a previous study [9], as follows: B1, within normal range (± 2 × standard deviation); B2, the field of BSV reaches at least 20 degrees superiorly, 40 degrees inferiorly, and 30 degrees horizontally; B3, a smaller field of BSV than B2 but includes primary gaze; B4, the field of BSV does not include primary gaze; B5, cannot obtain the field of BSV in any direction of gaze.

**Figure 1:**
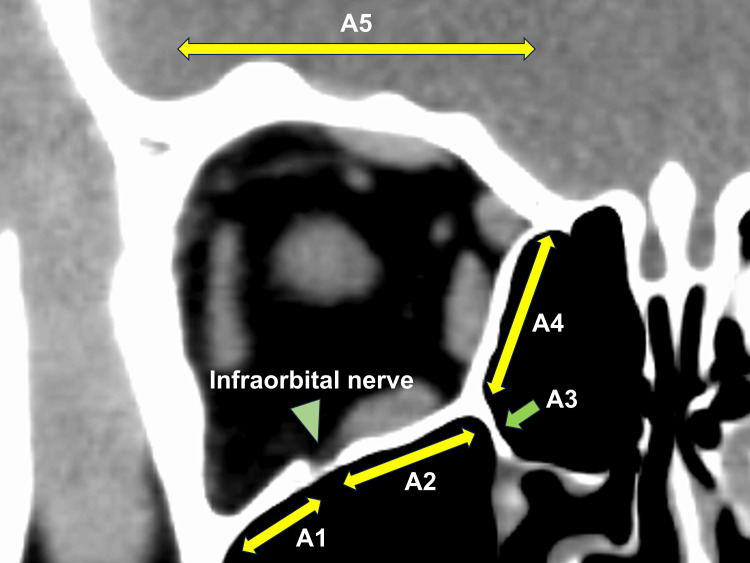
Classification of orbital fracture sites A1, the orbital floor lateral to the infraorbital nerve (green arrowhead); A2, the orbital floor medial to the infraorbital nerve (green arrowhead); A3, the inferomedial orbital strut (green arrow); A4, the medial orbital wall; A5, the orbital roof.

Axial and coronal orbital computed tomographic (CT) images with bone and soft tissue window algorithms were obtained from all patients. Orbital fracture sites and entrapped orbital soft tissues were examined. Orbital fracture sites were classified as follows, based on previous studies [8,10]: the orbital floor lateral to the infraorbital nerve (area 1, A1); the orbital floor medial to the infraorbital nerve (A2); the inferomedial orbital strut (A3); the medial orbital wall (A4); and the orbital roof (A5) (Figure 1). Entrapped orbital soft tissues in cases with trapdoor orbital fracture included the extraocular muscles and orbital fat.

The study period was divided into the pre-COVID-19 pandemic period from March 2017 to March 2020 and the period of the COVID-19 pandemic from April 2020 to April 2023, both of which were three years and one month in duration. During the COVID-19 pandemic, the Japanese government declared a state of emergency four times (first: April 7, 2020, to May 25, 2020; second: January 8, 2021, to March 21, 2021; third: April 25, 2021, to June 20, 2021; and fourth: July 12, 2021, to September 30, 2021) and issued priority preventative measures twice (first: April 5, 2021, to September 30, 2021; and second: January 9, 2022, to March 21, 2022). Even during the other periods of the COVID-19 pandemic, activities of people living in Japan had been restricted until the legal status of COVID-19 was subdued to “Class 5 (as common infectious diseases such as seasonal influenza)” on May 8, 2023.

Patient age was expressed as means ± standard deviations. Patient age was compared between patients consulted in the pre-COVID-19 pandemic (pre-COVID-19 pandemic group) and those consulted in the COVID-19 pandemic (COVID-19 pandemic group) using the Student’s t-test. A chi-square test was employed to compare the categorical variables between the groups. All statistical analyses were performed using Statistical Package for the Social Sciences (SPSS™) version 26 software (IBM Japan, Tokyo, Japan). Two-tailed P values < 0.05 were deemed to indicate statistical significance.

## Results

Data on patient characteristics and clinical findings are shown in Tables 1-2. We included 226 sides from 225 patients of pure orbital fracture aged ≤ 18 years (mean age, 12.7 ± 4.1 years; 177 males and 48 females). All except one patient sustained a unilateral orbital fracture. One of the patients was previously reported [11].

**Table 1:** Data on patient characteristics M, male; F, female; R, right; L, left *Statistical comparison using chi-square test.

Items	Total	Pre-COVID-19 pandemic	COVID-19 pandemic	P value
Number of patients/sides	225/226	114/115	111/111	-
Number of patients aged ≥ 7 years	202	102	100	-
M/F	177/48	94/20	83/28	0.193^*^
R/L	105/121	54/61	51/60	-
Causes of injury	-	-	-	-
Sports	137	74	63	0.610^*^
Assault	11	6	5	
Fall	28	12	16	
Traffic accident	11	4	7	
Others	38	18	20	
Causes of injury in patients aged ≥ 7 years	-	-	-	-
Sports	134	73	61	0.451^*^
Assault	11	6	5	
Fall	20	7	13	
Traffic accident	10	4	6	
Others	27	12	15	
Outdoor/indoor sports	50/87	26/48	24/39	1.000^*^

**Table 2:** Data on clinical findings IR, inferior rectus; MR, medial rectus; IO, inferior oblique; BSV, binocular single vision *Statistical comparison using chi-square test.

Items	Total	Pre-COVID-19 pandemic	COVID-19 pandemic	P value
Fracture sites	-	-	-	-
A1	2	0	2	0.056^*^
A2	127	62	65	
A4	55	27	28	
A1+A2	5	1	4	
A1+A2+A4	1	0	1	
A1+A4	3	3	0	
A1+A2+A3+A4	1	1	0	
A2+A4	21	11	10	
A2+A3+A4	6	5	1	
A5	5	5	0	
Entrapped orbital soft tissue	-	-	-	-
Orbital fat	109	55	54	0.688^*^
IR	20	9	11	
MR	3	1	2	
IR+IO	1	0	1	
Infraorbital nerve hypoesthesia	-	-	-	-
Presence	26	11	15	0.408^*^
Absence	200	104	96	
Preoperative field of BSV	-	-	-	-
B1	61	34	27	0.878^*^
B2	60	28	32	
B3	48	23	25	
B4	15	8	7	
B5	28	14	14	
Number of patients who underwent surgery	130	60	70	0.108^*^

The most common cause of injury was sports (137 sides, 60.6%), and outdoor sports were more common as causes of injury (87 sides, 63.5%), compared to indoor sports (50 sides, 36.5%). The most common fracture site was A2 (127 sides, 56.2%), followed by A4 (55 sides, 24.3%) and A2+A4 (21 sides, 9.3%). Trapdoor orbital fracture was observed on 133 sides (58.8%), and only orbital fat was entrapped on most of the sides (109 sides, 82.0%). Infraorbital nerve hypoesthesia was absent on 200 sides (88.5%). The field of BSV examined on the first visit was B2 or better in 121 patients (53.8%). Orbital fracture was reduced on 130 sides (57.5%).

The results of the statistical comparison between the groups are in Tables 1-2. Patients age (12.9 ± 4.2 years vs. 12.5 ± 4.1 years, P = 0.423) or male-to-female ratio (P = 0.193) was not significantly different between the groups. The most common cause of injury was sports in both groups, and the ratio of causes of injury (P = 0.610) or that between outdoor and indoor sports (P = 1.000) was not statistically different between the groups. Although we additionally compared the ratio of causes of injury in patients aged ≥ seven years because sports injuries increase as children achieve school age [12], the difference was not statistically significant (P = 0.451). The ratio of orbital fracture sites tended to be different between the groups (P = 0.056). Most sides with trapdoor orbital fracture showed only orbital fat entrapment in both groups (P = 0.688), and most sides lacked infraorbital nerve hypoesthesia in both sides (P = 0.408). The ratio of the field of BSV examined on the first visit was not different between the groups (P = 0.878), and the percentage of patients who underwent fracture reduction was not significantly different (P = 0.108).

Moreover, we compared the number of patients consulted with our service per diem and the ratio of causes of injury between the period during a state of emergency and/or priority preventative measures, in the COVID-19 pandemic other than the period of a state of emergency and/or priority preventative measures, and pre-COVID-19 pandemic. Although the daily rate of pediatric orbital fracture cases was lowest in the period during the country’s state of emergency with priority preventative measures, the difference was not statistically significant (Table 3) (P = 0.911). The ratio of causes of injury was not significantly different among the three periods (P = 0.530). To further go into detail, the daily rate of pediatric orbital fracture cases referred to our service was lowest in the period of the first state of emergency (Table 4). Although the case rate during the third state of emergency was the second lowest, the case rate in the other periods seemed to be similar to the situation before the COVID-19 pandemic. The difference in the daily rate was not statistically significant among the periods (P = 0.585).

**Table 3:** Comparison of the number of patients consulted with us/day and ratio of causes of injury among three periods *Statistical comparison using chi-square test.

Items	Pre-COVID-19 pandemic	COVID-19 pandemic	A state of emergency/priority preventative measures	P value
Number of patients	114	76	35	-
Daily rate (number/day)	0.1011	0.1012	0.938	0.911^*^
Causes of injury	-	-	-	-
Sports	74	47	16	0.530^*^
Assault	6	3	2	
Fall	12	11	5	
Traffic accident	4	3	4	
Others	18	12	8	

**Table 4:** The daily rate of the number of patients who consulted with our service in each period of the state of emergency/priority preventative measures

Periods	Number of patients	Daily rate
First state of emergency	2	0.0408
Second state of emergency	7	0.0958
Third state of emergency	4	0.0702
Fourth state of emergency	8	0.0988
First priority preventative measures	17	0.0949
Second priority preventative measures	10	0.1389

## Discussion

During the COVID-19 pandemic, the Japanese government declared a state of emergency four times: the first period from April 7 to May 25, 2020; the second period from January 8 to March 21, 2021; the third period from April 25 to June 20, 2021; and the fourth period from July 12 to September 30, 2021. Priority preventative measures were also imposed twice on April 5 to September 30, 2021, and on January 9 to March 21, 2022. Throughout the COVID-19 pandemic, activities of people living in Japan had been restricted until the legal status of COVID-19 was subdued to “Class 5 (as common infectious diseases such as seasonal influenza)” on May 8, 2023. This was the time when the schools resumed operations and the students were allowed to go to class physically.

When Japan declared the very first state of national emergency from April 7 to May 25, 2020, it was the time when people were overwhelmed with fear of the morbidity and mortality that the virus has brought upon those infected. Parents, especially those with young children, strongly abided by the restrictions imposed by the government in the hopes of protecting their children from transmitting the deadly virus. This has resulted in the initial decrease in hospital consultations, which may probably be due to the reduction of traumatic ocular injuries. As the country witnessed improved COVID-19 conditions, the restrictions were slowly lifted and children were allowed to play or go outside, causing a subsequent increase in the patients consulting for ocular injuries from sports, physical assault falls, traffic accidents, and other recreational activities. Despite the declaration of a second, third, and fourth state of emergency, and even when data was adjusted to analyze the incidence of blowout fractures in older children seven years old and above, in consideration of the fact that more children engage in sports as they become older, there was still no statistically significant difference in the pre-COVID group and the COVID group. These findings are surprisingly contradictory to the well-disciplined nature of the Japanese people. On the other hand, a possible explanation is that the children may have found ways to engage in sports and other recreational activities while observing social distancing measures. Another reason is the possibility of having more referrals from surrounding hospitals that are incapable of accommodating the patients due to the lack of manpower or specialists to manage the cases.

Although the Japanese children were maintained on online classes during the pandemic and in-person classes did not resume until the government considered COVID-19 completely nonfatal and comparable to the seasonal flu on May 8, 2023, the incidence of pediatric patients diagnosed with orbital fractures were surprisingly similar to the pre-pandemic conditions. Furthermore, in this study, sports-related injuries, specifically from outdoor sports, were still the most common cause of pediatric orbital fracture during the COVID-19 pandemic, much like before the pandemic even occurred. This is probably due to the fact that these children, after being deprived of recreational activities for more than a month, were very eager to engage in sports and other recreational activities. With regards to outdoor sports, there is also the perception of a lower risk of infection in open-spaced areas as compared to indoor spaces. Moreover, strict lockdown measures were not enforced, thereby maintaining the same level of risk for traumatic injuries from other related causes, such as falls, physical assaults, and traffic accidents, as well in these children.

Similar findings were presented in a study conducted in the Japanese Red Cross Kitami Hospital, wherein pediatric trauma-related hospitalization did not decrease in 2020 and fractures acquired outside the patients’ homes were the most common [2]. A study in Italy has also found that there was no statistically significant difference in the incidence of maxillofacial fractures between the pre-pandemic and pandemic periods [13]. Although traffic accidents and sports-related trauma declined during the pandemic, it was interesting to note a significant increase in trauma cases caused by physical assault. Giovannetti et al. explained how the nationwide restrictions may have led to a negative psychological impact on society [13], which was relevant to many other nations that underwent these strict measures to control the spread of COVID-19.

The most common fracture site seen in the pediatric patients in this study was on the maxillary bone component of the inferior orbital wall, classified as A2, and was followed by the ethmoid bone in the medial orbital wall, classified as A4. Considering the mechanism of orbital blowout fracture, damage to these locations in the bony orbital walls can indeed be found in sports injuries. A strong impact to the orbital rim from an object, such as a ball, or another human will cause a direct compression or “buckling force” on the orbital floor and medial orbital wall [8,14], thereby resulting in fractures at the A2 or A4 sites. Trapdoor orbital fractures are also most commonly found in children due to the characteristic elasticity of pediatric orbital bones [9,15,16]. With age, bones become more brittle and, therefore, form comminuted fractures instead of snapping back [17].

There are limitations to consider in this study. Since the study was conducted in a single institution and only included patients residing in Aichi, Mie, and Gifu prefectures, the possibility of sample selection bias exists and the results presented may have limited applicability to the general population in Japan. Furthermore, the COVID-19 situation in surrounding hospitals may have resulted in the referral of pediatric orbital fracture cases to our institution, consequently increasing the rate of patient consults.

## Conclusions

This retrospective cohort study has found no significant difference in the incidence of pediatric orbital fracture cases before and during the COVID-19 pandemic. The initial decrease in the rate of patient consultations during the first state of emergency period was probably due to the strict implementation of social distancing and restrictions imposed by the government, coupled with fear of infection from the people. The impression that Japanese people are very courteous and obedient may not automatically translate to a decrease in the incidence of orbital fractures among the Japanese population. Despite the restrictions brought about by the COVID-19 pandemic, the risk for orbital fractures in children remains unchanged.

## References

[1] Sekine I, Uojima H, Koyama H (2020). Impact of non-pharmaceutical interventions for the COVID-19 pandemic on emergency department patient trends in Japan: a retrospective analysis. Acute Med Surg.

[2] Maruo Y, Ishikawa S, Oura K (2022). The impact of the coronavirus disease 2019 pandemic on pediatric hospitalization in Kitami, Japan. Pediatr Int.

[3] Pellegrini M, Roda M, Di Geronimo N, Lupardi E, Giannaccare G, Schiavi C (2020). Changing trends of ocular trauma in the time of COVID-19 pandemic. Eye (Lond).

[4] Spallaccia F, Vellone V, Colangeli W, De Tomaso S (2022). Maxillofacial fractures in the province of Terni (Umbria, Italy) in the last 11 years: impact of COVID-19 pandemic. J Craniofac Surg.

[5] Wu C, Patel SN, Jenkins TL, Obeid A, Ho AC, Yonekawa Y (2020). Ocular trauma during COVID-19 stay-at-home orders: a comparative cohort study. Curr Opin Ophthalmol.

[6] Halawa OA, Friedman DS, Roldan AM, Zebardast N (2023). Changing trends in ocular trauma during the COVID-19 pandemic in the USA. Br J Ophthalmol.

[7] Agrawal D, Parchand S, Agrawal D, Chatterjee S, Gangwe A, Mishra M, Sahu A (2021). Impact of COVID-19 pandemic and national lockdown on ocular trauma at a tertiary eye care institute. Indian J Ophthalmol.

[8] Takahashi Y, Nakakura S, Sabundayo MS, Kitaguchi Y, Miyazaki H, Mito H, Kakizaki H (2018). Differences in common orbital blowout fracture sites by age. Plast Reconstr Surg.

[9] Takahashi Y, Sabundayo MS, Miyazaki H, Mito H, Kakizaki H (2018). Orbital trapdoor fractures: different clinical profiles between adult and paediatric patients. Br J Ophthalmol.

[10] de Silva DJ, Rose GE (2011). Orbital blowout fractures and race. Ophthalmology.

[11] Lee PA, Kono S, Kakizaki H, Takahashi Y (2022). Entrapment of the inferior oblique and inferior rectus muscles in orbital trapdoor fracture. Orbit.

[12] Sirichai P, Anderson PJ (2015). Orbital fractures in children: 10 years' experience from a tertiary centre. Br J Oral Maxillofac Surg.

[13] Giovannetti F, Lupi E, Di Giorgio D (2022). Impact of COVID19 on maxillofacial fractures in the province of L’Aquila, Abruzzo, Italy. Review of 296 patients treated with statistical comparison of the two-year pre-COVID19 and COVID19. J Craniofac Surg.

[14] Kono S, Vaidya A, Takahashi Y (2023). Mechanisms of development of orbital fractures: a review. Ophthalmic Plast Reconstr Surg.

[15] Phan LT, Jordan Piluek W, McCulley TJ (2012). Orbital trapdoor fractures. Saudi J Ophthalmol.

[16] Prasad C, Arulmozhi M, Balaji J, Nisha MP (2020). White-eyed blowout fracture. Ann Maxillofac Surg.

[17] Young SM, Kim YD, Kim SW, Jo HB, Lang SS, Cho K, Woo KI (2018). Conservatively treated orbital blowout fractures: spontaneous radiologic improvement. Ophthalmology.

